# Whole-Person, Whole-Team Approach to Quality Improvement: Why Person-Centered Care Matters

**DOI:** 10.1093/geroni/igaa057.855

**Published:** 2020-12-16

**Authors:** Ryann Engle, A Lynn Snow, Valerie Clark, Shibei Zhao, Christopher Gillespie, Christine Hartmann

**Affiliations:** 1 VA Boston Healthcare System, Boston, Massachusetts, United States; 2 Tuscaloosa VA Medical Center, Tuscaloosa, Alabama, United States; 3 Edith Nourse Rogers Memorial Veterans Hospital, Bedford, Massachusetts, United States

## Abstract

The Department of Veterans Affairs (VA) began its culture transformation journey in 2006, supporting its nursing homes in providing high-quality, person-centered care in person-centered environments. We implemented a quality improvement intervention to support frontline staff from low-performing VA nursing homes in providing high-quality care using a whole-person, whole-team approach. The intervention consisted of a bundle with four components: 1) specialized frontline staff huddles that encouraged high-quality frontline staff communication and collaboration, 2) micro-root cause analyses and targeted interventions to promote resident sleep and reduce resident falls through individualized care, 3) in-depth frontline conversations regarding residents’ distress behaviors and mobility, and 4) targeted, team-based, person-centered performance improvement projects. The intervention was implemented at 8 low-performing VA nursing homes (August 2018 - April 2019) via in-person and virtual sessions and facilitated through CLC-based champions and intervention team-based coaches. We monitored the intervention’s impact using pre-post Centers for Medicare and Medicaid Services quality star ratings. We also conducted 17 post-intervention interviews with key informants at 7 participating nursing homes and conducted a content analysis of the data. Pre intervention, all 8 nursing homes had a history of being 1 or 2 stars in overall quality. Post intervention, 3 homes increased 1 star; 1 home increased 2 stars; 2 homes increased 3 stars; 2 homes increased 4 stars. Post intervention, participants perceived improved delivery of person-centered care (e.g., providing individualized sleep hygiene, de-implementing alarms). Our findings suggest a whole-person, whole-team intervention can effectively and efficiently improve both person-centered care and care quality.

